# Ginsenoside Rg1 Alleviates Hepatic Ischemia-Reperfusion Injury in Mice via Activating ER*α*-Regulating YAP Expression

**DOI:** 10.1155/2021/6486109

**Published:** 2021-09-29

**Authors:** Kehui Zhang, Jiacheng Li, Yong Li

**Affiliations:** ^1^Shanghai Municipal Hospital of Traditional Chinese Medicine, Shanghai University of Traditional Chinese Medicine, Shanghai 200071, China; ^2^Shanghai University of Traditional Chinese Medicine, Shanghai 200071, China

## Abstract

**Objective:**

To verify whether ginsenoside Rg1 alleviates liver hepatic ischemia-reperfusion injury (IRI) in mice by upregulating the expression of Yes-associated protein (YAP) through estrogen receptor alpha pathway.

**Methods:**

The whole hepatic IRI model and the local (70%) hepatic IRI model were established, respectively. The whole hepatic IRI model was used to observe the survival curve of mice, and the mouse models with 70% hepatic IRI were used to explore the mechanism of liver injury about Rg1 in hepatic IRI. Wild-type C57BL/6 mice were randomly divided into some groups: (1) the whole hepatic IRI model group: the survival rate of mice was observed at 0, 30, 60, 90, and 120 min after ischemia and Rg1 intervention (90 min after ischemia), with 10 mice in each group, and (2) the 70% hepatic IRI model group: sham operation group, I/R model group, verteporfin (VP) group, doxycycline (Doxy) group, 17*β*-estradiol (E2) group, clomiphene (Clom) group, and Rg1 group with 6 mice in each group. The level of serum alanine aminotransferase (ALT) was measured by enzyme labeling instrument, the degree of liver injury was analyzed after hematoxylin-eosin (HE) staining, and the function of mitochondria was detected in fresh liver tissue, including mitochondrial membrane potential with JC-1 (5,5′,6,6′-tetrachloro1,1′,3,3′-tetramethylbenzimidazolylcarbocyanine iodide), adenosine triphosphate (ATP), and mitochondrial reactive oxygen species (ROS), and the expression of YAP and estrogen receptor alpha (ER*α*) genes and proteins were detected by real‐time reverse‐transcriptase polymerase chain reaction (RT-PCR) and Western blot.

**Results:**

The whole hepatic IRI model showed that the survival rate of mice decreased with the prolongation of ischemia time. IRI model mice showed mitochondrial damage, JC-1 red/green fluorescence value and ATP significantly decreased, and ROS production increased; in comparison, in the Doxy and E2 intervention group, JC-1 red/green fluorescence value and ATP production increased and ROS downregulated, indicating that mitochondrial function returned to normal. The level of serum ALT showed that the liver enzyme increased with the time of reperfusion and decreased gradually after 6 hours. The results of Western blot and PCR showed that the expression of YAP and ER*α* showed the same trend. The IRI model mice were observed after 90 minutes of ischemia and 6 hours of reperfusion. Compared with the corresponding sham group, the expression of YAP in the liver tissue of the Doxy group, E2 group, and Rg1 intervention group increased, and the expression of ER*α* in the E2 group and Rg1 group increased. HE staining showed that a large number of inflammatory cell infiltration could be seen in the liver tissue of the model group, but it decreased in the Doxy and E2 intervention groups.

**Conclusion:**

Ginsenoside Rg1 exerts an estrogenic effect by activating ER*α*, upregulating the expression of YAP, reducing liver oxidative stress injury, and inhibiting mitochondrial injury to protect the liver from ischemia-reperfusion injury in mice.

## 1. Introduction

IRI is an exogenous non-antigen-dependent local inflammatory reaction caused by hypoxic stress, which often occurs in orthotopic liver transplantation (OLT), vascular embolism, hemorrhagic shock, acute ischemic trauma, drug-induced liver injury, and other diseases. The repair of liver injury mediated by IR is the difficulty in the treatment of these diseases. At present, the clinical treatment of hepatic IRI mainly focuses on reducing ischemic injury, controlling reperfusion conditions, improving ischemic tissue metabolism, antioxidation, scavenging free radicals, and so on. Some studies have confirmed that estrogen has a protective effect on liver injury in IR. Other studies have confirmed that Hippo-YAP pathway plays a key role in liver IR-mediated hepatocyte injury and liver fibrosis, and there is a mutual regulation between estrogen and Hippo-YAP signal pathway. In this study, we established a mouse model of hepatic IRI. Through drug preconditioning, we investigated the expression of YAP and the protective effects of estrogen on liver injury and liver fibrosis and studied the mechanism of Rg1 alleviating hepatic IRI by upregulating the expression of YAP by ER*α*.

## 2. Materials and Methods

### 2.1. Experimental Animals

Wild-type C57BL/6 male mice of 7–8 weeks were selected. Mice can freely obtain chow and water before and after surgical operation.

#### 2.1.1. Model of Total Hepatic Ischemia (Survival Study)

Mice were anesthetized by intramuscular injection of ketamine (83.3 mg/kg rats) and acetopromazine (0.03 mg/kg rats). After midline laparotomy, the triad of the portal vein was exposed and all structures of the left and middle hepatic lobe (hepatic artery, portal vein, and bile duct) were occluded with the soft vascular clamp. The ischemic period was 30, 90, and 120 min, respectively. Retaining the perfusion of the remaining liver can prevent mesenteric venous congestion during ischemia. After the continuous ischemia, the abdominal cavity was reopened, the vascular clamp was quickly removed, and the blood flow of the ischemic liver was restored. The gradual recovery of the liver from white to bright red in the ischemic area indicated the success of reperfusion. The nonischemic lobe (right lobe and caudate lobe) was removed immediately after reperfusion. Then, suture the abdominal muscle and skin layer by layer to close the abdominal cavity. The survival of animals depends entirely on the remaining 70% of the liver damaged by ischemia. Animals that survive seven days after surgery are considered survivors. The mice with 90 minutes of ischemia were injected Rg1 (30 mg/kg) intravenously 1 h before surgery.

#### 2.1.2. Model of Partial (70%) Hepatic Ischemia

Anesthetized mice were subjected to 70% ischemia for 90 minutes as described above without removing the remaining liver lobes. There was no vascular occlusion in sham group mice. The mice were sacrificed at different periods after reperfusion; liver and serum samples were collected for analysis. In the treatment groups, mice were injected with doxycycline (Doxy, YAP activator, 5 mg/kg), verteporfin (VP, YAP inhibitor, 0.6 mg/kg), 17*β*-estradiol (E2, estrogen agonist, 3 mg/kg), or clomiphene (Clom, estrogen blocker, 1 mg/kg) intravenously 1 h before surgery. In the course of the experiment, the disposal of mice was carried out in accordance with the relevant provisions of the regulations on the Administration of Experimental Animals promulgated by the Science and Technology Commission of the People's Republic of China, which met the requirements of animal ethics management.

### 2.2. ALT Detection

ALT kits (Nanjing Jiancheng Biology Research Institute Co., Ltd.) were used to detect the serum level. After adding reagents in turn according to the instructions, the optical density (OD) value of each hole was determined by enzyme labeling instrument (wavelength: 510 nm). The absolute OD value = the OD value of the measured hole − the OD value of the control hole. Compared with the standard curve, the corresponding ALT activity unit was calculated.

### 2.3. ATP Detection

According to the determination instructions of the ATP detection kit (Beyotime Institute of Biotechnology, China), the ATP level of liver tissue homogenate was measured with the test kit.

### 2.4. JC-1 Detection

JC-1 probe (Beyotime Institute of Biotechnology, China) was carried out for mitochondrial membrane potential detection. The mitochondria of liver tissue were isolated; then, the purified mitochondria were resuspended and mixed with JC-1 staining solution and after that added to the 96-well plate to detect by fluorescence enzyme labeling instrument.

### 2.5. MitoSOX Red Mitochondrial Superoxide Indicator Detection of Liver Tissue

MitoSOX Red Mitochondrial Superoxide Indicator (Yisheng Biological Company, China), a new type of mitochondrial fluorescent probe, can specifically target mitochondria to selectively detect superoxides in mitochondria. Firstly, separate the liver tissue mitochondrial: the weight of cut tissue was about 20 mg and placed in 1.5 ml centrifuge tube, and the tissue was washed once or twice with precooled PBS. Put the tissue in an icebox and cut it into as small pieces as possible with scissors. Add 500 *μ*l precooled mitochondrial separation reagent A to grind fully (60HZ, 60S) for 3 times, absorb 300 *μ*l of the supernatant, transfer it to another centrifugal tube, and centrifuge at 4°C, 12,000 g for 10 minutes. The supernatant is carefully removed and the precipitation is the isolated mitochondria. 40 ul mitochondrial storage solution was added to resuscitate the mitochondria. Second, 300 *μ*l MitoSOX was added to a 96-well plate, and then 10 *μ*l purified mitochondria was added to each well, shaken and mixed well, and incubated in incubator at 37°C for 30 minutes. At last, the fluorescence intensity was detected by fluorescence microplate: Ex/Em = 510/580 nm.

### 2.6. HE Staining of Pathological Sections

Liver tissues taken from mice in each group were paraffin-embedded and stained with HE according to the standard procedure. The staining and morphology of the nucleus or cytoplasm were observed under microscope.

### 2.7. Real-Time PCR

Trizol (Invitrogen, USA) was applied for total RNA extraction. Then, reverse transcription and quantitative real-time PCR were performed with PrimeScript® RT reagent kit (Takara, Japan) and SYBR Premix Ex Taq (TaKaRa, Japan), respectively. The production of primers was supported by Shanghai Shenggong Biology Co., Ltd. Expression levels of genes normalized to *β*-actin levels in each sample were calculated according to the 2^−ΔΔCt^ method. The primer sequences are shown in Table 1.

### 2.8. Western Blotting

Proteins from liver tissue are electrophoretic on sodium dodecyl sulfate (SDS) polyacrylamide gel and then transferred to polyvinylidene fluoride (PVDF) membrane. YAP, ER*α*, and *β*-actin monoclonal antibodies (mAbs) (Cell Signaling Technology, Danvers, MA) were used as probes. After sealing with 5% skim milk, the membrane is incubated with the corresponding primary antibody, then incubated with the corresponding secondary antibody, and finally exposed.

### 2.9. Statistical Methods

The data were analyzed and plotted by SPSS22.0, GraphPad 5.0, and the data were expressed by mean ± SEM (Standard Error of Mean). Analysis of variance (ANOVA) was used for comparison among groups, and one-way analysis of variance (one-way ANOVA) was used for comparison between groups. A bilateral test was used. *P* < 0.05 is considered to be statistically significant.

## 3. Results

### 3.1. Survival Analysis of Total Hepatic Ischemia-Reperfusion model and Rg1 Ameliorated Hepatic IRI

**Figure 1:**
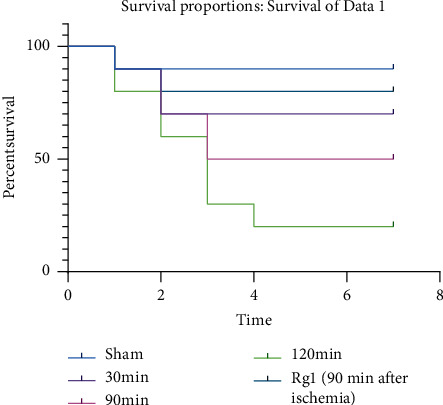
Survival analysis of total hepatic ischemia-reperfusion model and Rg1 ameliorated liver IRI. WT mice were subjected to sham operation or total hepatic ischemia-reperfusion for 30 min, 90 min, and 120 min, respectively. Animals surviving for 7 days after surgery are considered survivors. The mice subjected 90 min ischemia and pretreated with Rg1to observed the 7-day survival rate. *n* = 10, *P* ≤ 0.05.

WT mice were subjected to total hepatic IR for 30 min, 90 min, and 120 min, respectively. Animals surviving for 7 days after surgery are considered survivors; then, the survival curve was constructed. It was found that, with the extension of hepatic ischemia time, the survival rate of mice was shorter, and the survival rate was inversely proportional to the time of ischemia. We selected the 90 min model of liver ischemia and pretreated the mice with ginsenoside Rg1. The results showed that the survival rate of mice in the IR model group was higher than that in the simple IR model group, which was statistically significant (Figure 1, *P* ≤ 0.05).

### 3.2. Liver Injury and YAP Expression Profile in Murine Hepatic IRI

**Figure 2:**
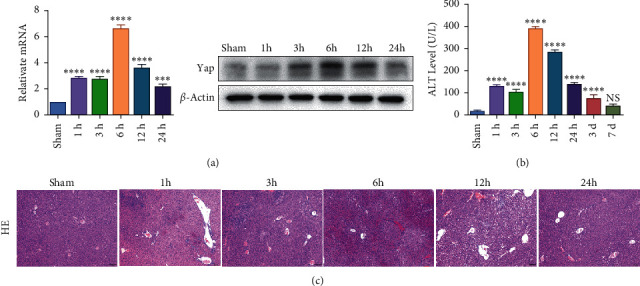
Liver injury and YAP expression profile in murine hepatic IRI mice subjected to 90 minutes of ischemia and then sacrificed after 1 h, 3 h, 6 h, 12 h, and 24 h after reperfusion. (a) Dynamic changes of Yap expression in mouse liver were detected by PCR and WB, *n* = 3. (b) Dynamic changes of ALT level, *n* = 6. (c) HE staining (×200; scale bar: 50 *μ*m). Data are expressed in mean ± SEM; ^∗∗∗^*P* < 0.001, ^∗∗∗∗^*P* < 0.0001, versus sham group; NS, nonsignificant.

To investigate whether IR triggered the expression of endogenous YAP gene in the liver of mice subjected to 90 minutes of warm ischemia, then the mice were sacrificed after 1 h, 3 h, 6 h, 12 h, and 24 h after reperfusion. The liver tissues of mice were collected for PCR and WB detection, and the dynamic changes of YAP expression were observed. Unlike in sham controls, YAP level increased at 1 hour after ischemia, peaking at 6 h after reperfusion and decreasing thereafter (Figure 2(a)). In addition, we determined whether IR triggered the liver injury in mice livers after 90 min of warm ischemia, followed by killed mouse at 1 h, 3 h, 6 h, 12 h, 24 h, 3 d, and 7 d after reperfusion. The serum of peripheral blood was collected to detect the level of ALT. The results showed that the level of ALT reached the peak at 6 h after reperfusion and then decreased gradually (Figure 2(b)). Unlike the sham group, liver tissue HE staining showed that the liver tissue of IR mice showed widespread hemorrhage, more inflammatory cell infiltration, severe lobular edema, and congestion/hepatocellular necrosis (Figure 2(c)). After 1 h of reperfusion, hepatocytes suffered wide congestion. As the reperfusion time is continuously extended, the liver lobular structure was destroyed, inflammatory infiltration increased, as well as hepatocyte necrosis, and decreased thereafter.

### 3.3. YAP Activation Attenuated Oxidative Stress and Liver Injury in Hepatic IRI

IRI in ischemia and hypoxia injury lead to the occurrence of mitochondrial permeability transition (MPT), the increase of ROS, mitochondrial DNA mutation, decrease of transmembrane potential, ATP energy exhaustion, and eventually hepatocytes necrotic [1]. To investigate the relationship between YAP activation and IR mice, we treated IR mice with YAP agonists and inhibitors to observe the recovery of liver injury in mice. To explore the relationship between YAP activation and liver injury in IR mice, we treated IR mice with YAP agonists and inhibitors to observe the recovery of liver injury in mice. Experimental grouping is as follows: (1) Sham group; (2) IR model group; (3) Doxy group; and (4) VP group.

**Figure 3:**
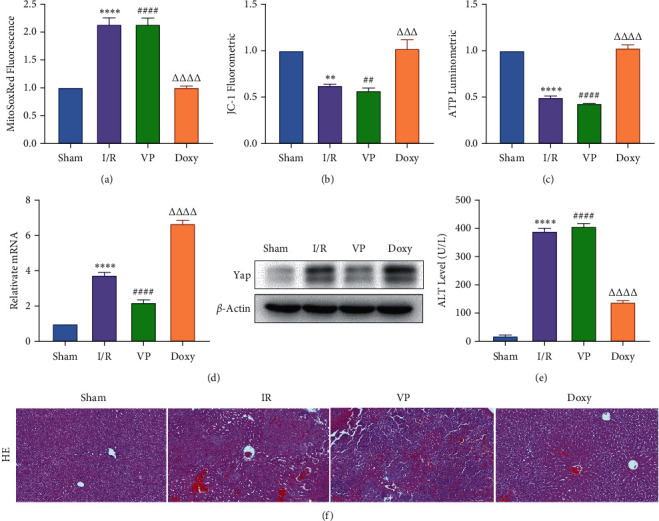
YAP activation attenuated oxidative stress and liver injury in hepatic IRI. (a) MitoSOX red mitochondrial superoxide indicator detection, *n* = 6. (b) Mitochondrial JC-1 fluorescence expression, *n* = 6. (c) ATP content, *n* = 6. (d) Relative mRNA and protein expression of Yap, *n* = 3. (e) The changes of serological ALT, *n* = 6. (f) HE staining (×200; scale bar: 50 *μ*m). Data are expressed in mean ± SEM; ^∗∗^*P* < 0.01, ^∗∗∗^*P* < 0.001, ^∗∗∗∗^*P* < 0.0001, versus sham group; ^##^*P* < 0.01, ^####^*P* < 0.0001, versus Doxy group; ^△△△△^*P* < 0.001, ^△△△△^*P* < 0.0001, versus IR group.

Compared with normal groups, IR mice can reduce the activity of mitochondrial ROS. After the YAP agonist intervention can reduce the generation of ROS, verteporfin exerted an opposite effect, indicating that the application of Doxycycline can be suppressed by hepatocyte oxidation (Figure 3(a)). The results of JC-1 mitochondrial fluorescence and ATP content detection showed that JC-1 membrane potential decreased and ATP production decreased compared with Sham group, while green fluorescence decreased compared with IR group after intervention with YAP agonist, and ATP level increased after intervention with doxycycline, indicating that YAP agonist has a protective effect on mitochondrial damage (Figures 3(b) and 3(c)). It is confirmed that YAP agonist has a protective effect on oxidative stress of hepatocyte mitochondria after hypoxia-reoxygenation in mouse liver. Moreover, RT-PCR and WB results disclosed that the application of YAP agonist activates the expression of YAP, the inhibitor reversed (Figure 3(d)). Compared with the IR group, after doxycycline intervention treatment, the peripheral serum ALT level of mice decreased (Figure 3(e)), liver pathological HE staining showed that liver inflammatory cell infiltration decreased. Unlike in Sham controls, liver tissue HE staining showed that the liver tissue of IR mice showed widespread hemorrhage, more inflammatory cell infiltration, severe lobular edema, and congestion/hepatocellular necrosis in the VP group (Figure 3(f)), confirming that YAP activation can alleviate liver injury.

### 3.4. ER*α* Expression Profile in Murine Hepatic IRI and ER*α* Activation Alleviates Liver IR Injury

**Figure 4:**
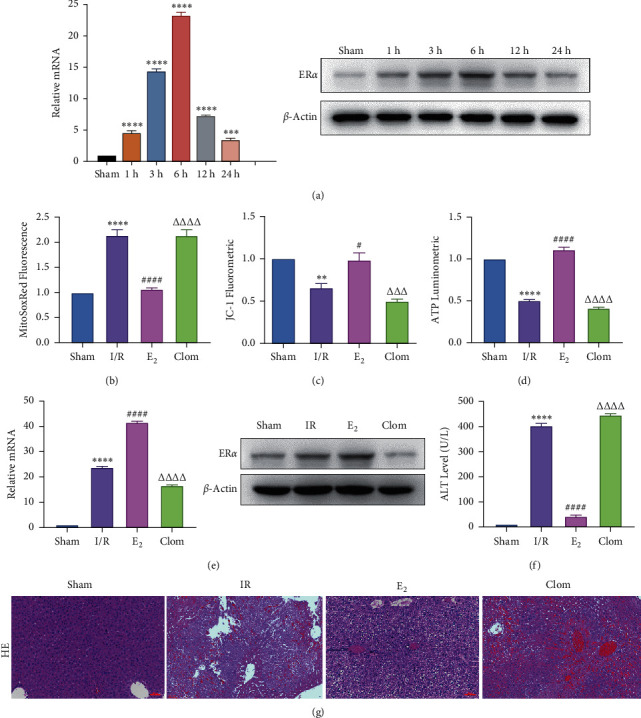
ER*α* expression profile in murine hepatic IRI; ER*α* activation alleviates liver IR injury. (a) Detection of ER*α* gene and protein level in IR mice at different time points after reperfusion, *n* = 3. (b) Mitochondrial ROS content, *n* = 6. (c) Mitochondrial JC-1 membrane potential of mouse liver, *n* = 6. (d) ATP content, *n* = 6. (e) Relative mRNA and protein expression of ER*α*, *n* = 3. (f) Serological ALT level, *n* = 6. (g) HE staining of liver tissue (×200; scale: 50 *μ*m). Data are expressed in mean ± SEM; ^∗∗^*P* < 0.01, ^∗∗∗∗^*P* < 0.0001, versus sham group; ^#^*P* < 0.05, ^####^*P* < 0.0001, versus IR group; ^△△△△^*P* < 0.001, ^△△△△^*P* < 0.0001, versus E2 group.

Estrogen has a protective effect on hepatic IRI in mice. In order to verify the relationship between estrogen, ER*α*, and liver IR, we observed the expression of ER*α* in liver tissue of mice at different reperfusion time points (1, 3, 6, 12, and 24 hours) after ischemic 90 min, and observed the liver injury of IR mice treated with estradiol and estrogen inhibitor clomiphene to observe the liver injury of IR mice. Experimental grouping is as follows: (1) Sham group; (2) IR model group; (3) Doxy (YAP agonist) group; and (4) VP (YAP inhibitor) group. The results revealed that the expression of ER*α* in mouse liver tissue increased and then decreased with the different duration of reperfusion, and the expression level at 6 h after reperfusion was the highest, which was the same as that of YAP, indicating that the ER*α* was in a state of protective elevation at the early stage of IR (Figure 4(a)). In the E2 group, the production of ROS decreased and Clom group reversed (Figure 4(b)). The results of JC-1 mitochondrial fluorescence explored that the membrane potential of JC-1 increased in the E2 group (Figure 4(c)), and the count of ATP also increased after the intervention of E2, which proved that estrogen had a protective effect on mitochondrial damage (Figure 4(d)). Consistently, the expression of mRNA and protein of ER*α* was increased after administration of estrogen receptor agonists (Figure 4(e)). The level of serum ALT in mice was detected 6 hours after reperfusion. The results showed that estradiol decreased the level of ALT, but clomiphene could not (Figure 4(f)). HE pathological staining detected that E2 intervention alleviated liver inflammatory cell infiltration, and more severe lobular edema, widespread hemorrhage, congestion/hepatocellular necrosis could be seen in the clomiphene group (Figure 4(f)). It is suggested that estrogen agonists can effectively alleviate IR-mediated inflammatory liver injury.

### 3.5. Ginsenoside Rg1 Activates Estrogen Receptor Alpha, Upregulates YAP Expression, and Alleviates IR-Mediated Liver Injury

**Figure 5:**
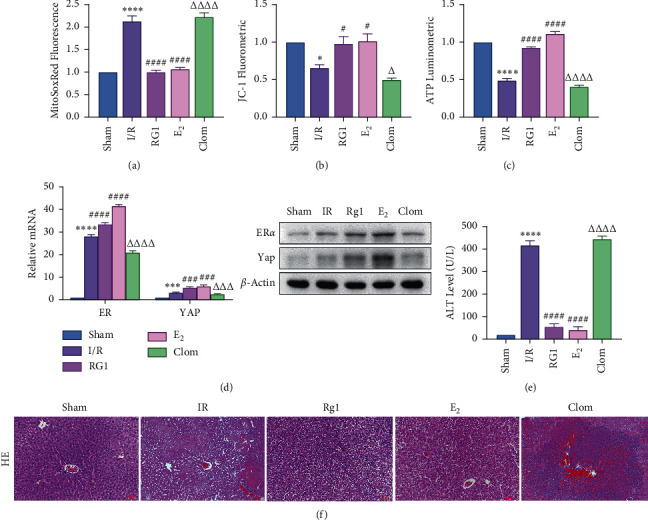
Ginsenoside Rg1 activates ER*α*, upregulates Yap expression, and alleviates IR-mediated liver injury. IR mice were subjected to 90 min of ischemia and reperfusion for 6 h. (a) Mitochondrial ROS content, *n* = 6. (b) The mitochondrial JC-1 membrane potential of mouse liver, *n* = 6. (c) The content of ATP, *n* = 6. (d) The expression of ER*α* and Yap at the level, *n* = 3. (e) The ALT level, *n* = 6. (f) HE staining of liver tissue (×200; scale: 50 *μ*m). Data are expressed in mean ± SEM; ^∗∗^*P* < 0.01, ^∗∗∗∗^*P* < 0.0001, versus sham group; ^#^*P* < 0.05, ^####^*P* < 0.0001, versus IR group; ^△△△△^*P* < 0.0001, versus E2 group.

To detect whether ginsenosides can activate ER*α* to exert an estrogen-like effect and upregulate the expression of YAP by activating ER*α* to alleviate liver injury after IR, we intervened in IR model mice with Rg1, E2, and clomiphene. Experimental groups is as follows: (1) Sham group; (2) IR group; (3) Rg1 group; (4) E2 group; and (5) Clom group. MitoSOX™ Red fluorescence detection showed that Rg1 could reduce the production of mitochondrial ROS, and the effect was the same as that of E2 (Figure 5(a)). The mitochondrial membrane potential and ATP content increased in the Rg1 pretreatment group, which confirmed that ginsenoside Rg1 could alleviate the oxidation and mitochondrial damage of hepatocytes in IR model mice (Figures 5(b) and 5(c)). Through the detection of PCR and WB, it was found that after the intervention of ginsenoside Rg1 and E2, the expression of ER*α* in the liver of IR mice was higher than that of the model group, and the expression of YAP showed the same trend, and the intervention of estrogen inhibitor could reduce that (Figure 5(d)). It is suggested that Rg1 can activate ER*α* and play an estrogen-like role. Moreover, the activation of ER*α* can affect the expression of YAP. Meanwhile, mice pretreated with Rg1 were resistant to liver IRI, demonstrated by reduced ALT levels and well-preserved hepatic architecture, without sinusoidal congestion, edema, and inflammatory cell infiltration (Figures 5(e) and 5(f)). In short, there was no significant difference in the therapeutic effect between the Rg1 intervention group and the E2 group. It is confirmed that Rg1 can play an estrogenic role, activate ER*α*, upregulate the expression of YAP, and play a protective role in liver injury after IR.

## 4. Discussion

Hepatocytes are directly damaged in the process of IRI. After hepatic ischemia, reductive substances are consumed, and anaerobic metabolism predominate. When ischemia and hypoxia occur in the liver, the transport of oxygen and nutrients is impaired, and the lack of oxygen as the terminal electron carrier of the mitochondrial respiratory chain will immediately interrupt the mitochondrial electron flow, resulting in respiratory chain dysfunction, increase in the intracellular NADH/NAD+ (nicotinamide adenine dinucleotide) ratio, termination of oxidative phosphorylation, and the depletion of cellular ATP. The respiratory chain of hepatocytes is interrupted, and the mitochondria that lose the supply of oxygen and glucose cannot produce enough ATP supply, which will lead to the production of ROS, and the accumulation of toxic substances, accelerated glycolysis, and increased lactic acid formation, resulting in hepatocyte damage [2]. In addition, the loss of oxidative phosphorylation substrate leads to the increase of mitochondrial permeability and the damage of mitochondrial membrane potential, especially in the reperfusion stage, which increases the production of ROS and results in further necrotic injury to hepatocytes [3]. Studies have confirmed that the intervention of ROS scavengers can effectively alleviate IR-mediated liver injury [4].

In the process of reperfusion, oxygen is reintroduced, which requires a large number of reducing substances, thereby producing excessive oxygen free radicals. Oxygen free radicals lead to direct cell damage and induce inflammation response, which result in the production of many cytokines, such as interleukin-1*β* (IL-1*β*), interleukin-2(IL-2), interleukin-8 (IL-8), and tumor necrosis factor-*β* (TNF- *β*) [5]. Pro-inflammatory cytokines and damaged hepatocytes release ROS. High-mobility group protein B1 (HMBG1) and damage-associated molecular pattern (DAMP) lead to neutrophil recruitment and Kupffer cell activation [6]. It can be seen that the main characteristics of hepatocyte death are hepatocyte necrosis induced by ROS and apoptosis mediated by congenital proinflammatory cytokines [7]. Our results also confirmed that the production of ROS in hepatocytes of IR model mice increased, the membrane potential of JC-1 and the content of ATP decreased, and a large number of inflammatory cells could be seen in the pathological sections of liver tissue. As a key downstream factor of Hippo signaling pathway, YAP plays a key role in ischemia-reperfusion-induced hepatocyte injury and liver fibrosis.

It has been found that, in the early stage of mouse IR, the level of YAP increases and YAP undergoes nuclear localization, indicating that YAP may play a role in the early stage of hepatic stellate cell (HSC) activation [8]. The activation of YAP protects hepatocytes threatened by IR to a great extent; on the contrary, YAP inhibition aggravates hepatocyte injury and promotes inflammation, HSC activation, and liver fibrosis [8]. Our experimental results also confirmed that the expression of YAP in mouse liver increased in the early stage of IR and decreased with the prolongation of reperfusion time, suggesting that YAP would have a protective increase in the early stage of the disease. The activation of YAP protects the liver from the threat of IR by promoting regeneration and induction of antioxidant genes while reducing oxidative stress, necrosis/apoptosis, and congenital inflammation [8]. Clinical studies have shown that the expression of graft YAP after human liver transplantation is negatively correlated with liver function and tissue injury [8]. We pretreated IR model mice with doxycycline and VP. It can be seen that the application of YAP agonists can lead to antioxidant stress, alleviate liver mitochondrial injury in mice, and exert the protection of IR liver injury. Estrogen and estrogen-like drugs exert their biological effects by activating the estrogen receptor and protect IR. The results showed that the level of serum ALT and the expression of TNF-*α* and NF-*κ*B in serum and tissue of IR mice pretreated with estrogen decreased, and the degree of liver injury could be alleviated [9]. Our results showed that, compared with the Sham group, the expression level of ER*α* in liver tissue of the IR group increased at the initial stage, which was proportional to the growth of reperfusion to some extent and consistent with the trend of YAP expression. The expression of ER*α* in the E2 group was higher than that in the IR group, while the expression of ER*α* in the Clom group decreased. The intervention of E2 can alleviate the oxidative stress injury of mitochondria in IR mice and protect liver function. The intervention of Clomiphene aggravates the liver injury, indicating that the increased expression of estrogen receptor alpha is beneficial to the recovery of liver injury after IR. YAP can regulate each other with estrogen receptor alpha. Pandey and Zhou et al. showed that stimulation of *G* protein-coupled estrogen receptor (GPER) can activate YAP and transcriptional coactivator with PDZ-binding motif (TAZ) by inducing dephosphorylation of YAP/TAZ and nuclear localization [6, 10]. Zhu [11] believed that YAP/TEAD, the nuclear effector of the Hippo pathway, was a cofactor of ER*α*. Mechanistically, studies have identified a direct interaction between Hippo and ER*α* signaling [12]. We intervened IR mice through E2 and clomiphene and found that the expression of YAP in the E2 group was higher than that in the Sham group and IR group, while the expression of YAP in the Clom group was significantly decreased, which confirmed that the activation of ER*α* could regulate the expression of YAP. Ginsenoside Rg1 has a phytoestrogenic effect [13]. It has been found that ginsenoside Rg can exert an estrogenic effect by rapidly inducing the formation of ER-containing signal bodies in MCF-7 cells or directly activating membrane-related ER and GPER [14].

Rg1 can protect mouse liver from IRI in many ways [15]. Our results also confirmed that the intervention of Rg1 can activate the activity of ER*α*, upregulate the expression of YAP, play a role similar to E2, and alleviate mitochondrial oxidative stress, membrane potential damage, and ATP production and is effective in the repair of liver injury after IR.

To sum up, ginsenosides can play an estrogenic effect by activating ER*α*, upregulating the expression of YAP, reducing liver oxidative stress injury, inhibiting mitochondrial membrane damage, increasing ATP content, and then protecting the liver from IRI in mice. Our research is only carried out in animal models, and there are limitations due to the lack of in vitro cell models and clinical application evidence. We will further explore the effects of Rg1 on the upstream and downstream factors of the Hippo-YAP signal pathway and hepatocyte necrosis and apoptosis, confirmed in vitro and supported by clinical evidence, and further clarify the mechanism of the effect of Rg1 on IR. This study is expected to provide a theoretical basis for ginsenoside Rg1 as a new drug for the prevention and treatment of hepatic IRI.

## Figures and Tables

**Table 1:** Sequences of primers (mice) used in real-time PCR.

	5′-primer	3′-primer
YAP	AAGACACTGCATTCTGAGTCC	GAATATCAATCCCAGCACAGC
ER*α*	AGGCGGCATACGGAAAGAC	GCTGTCACCTTCACCGTTCC
*β*-Actin	CTCCATCCTGGCCTCGCTGT	CTCCATCCTGGCCTCGCTGT

## Data Availability

The data used to support the findings of this study are included within the article.
